# Translating thyroid hormone into clinical practice: lessons learned from the *post-hoc* analysis on data available from the ThyRepair study

**DOI:** 10.3389/fendo.2024.1405251

**Published:** 2024-07-26

**Authors:** Constantinos I. Pantos, Konstantinos P. Grigoriou, Athanasios G. Trikas, Nikolaos A. Alexopoulos, Iordanis S. Mourouzis

**Affiliations:** ^1^ Department of Pharmacology, National and Kapodistrian University of Athens, Athens, Greece; ^2^ Department of Cardiology, ELPIS General Hospital of Athens, Athens, Greece; ^3^ Department of Radiology, IASO Hospital of Athens, Athens, Greece; ^4^ Cardiovascular Imaging Unit, Department of Radiology Athens Euroclinic, Athens, Greece

**Keywords:** thyroid hormone, myocardial infarction, reperfusion, cardiac remodeling, heart failure

## Abstract

**Background:**

Thyroid hormone (TH) appears to have a reparative action on the postinfarcted myocardium. This novel action was recently tested in a pilot, randomized, double-blind, placebo-controlled trial (ThyRepair). The present study performed a *post-hoc* analysis of data from the ThyRepair study to provide further insights into the novel actions of TH on the human postischemic myocardium.

**Methods:**

Data from 41 patients participating in the ThyRepair study (n = 20 placebo and n = 21 LT3) were included in the analysis. LT3 treatment started after stenting and continued intravenously for 48 h. All patients had cardiac magnetic resonance (CMR) at hospital discharge; left ventricular (LV) ejection fraction (LVEF%), LV end-diastolic volume index (LVEDVi; mL/m^2^), LV end-systolic volume index (LVESVi; mL/m^2^), infarct volume (IV), left ventricular mass index (LVMi) as edema index, and microvascular obstruction (MVO) were assessed. Patients were divided into two groups based on the median value of the IV: patients with IV ≤ 20% of the LV (group A) and patients with IV > 20% (group B). CMR measurements at discharge are expressed as mean ± SD.

**Results:**

In group A, the placebo and T3-treated groups had similar LVEF% (56.8 ± 10.2 vs. 52.2 ± 10.5), LVEDVi (90.9 ± 19.8 vs. 92.8 ± 14.5), and LVESVi (40.8 ± 18.2 vs. 44.9 ± 14.1) at discharge. In group B, LVEDVi and LVESVi were 112 ± 23.8 and 68.3 ± 21.5 for placebo vs. 91.8 ± 18.6 and 49.0 ± 14.0 for the T3-treated group, respectively, p < 0.05. LVEF% was significantly increased in the T3-treated group vs. placebo, 47.3 ± 6.5 vs. 39.9 ± 8.7, p < 0.05. In group B, CMR LVMi was lower in T3-treated patients vs. placebo but did not reach statistical significance (p = 0.1). MVO was 1.95 ± 2.2 in placebo vs. 0.84 ± 0.9 in the LT3-treated group, p = 0.15.

**Conclusion:**

The present study suggests that acute LT3 treatment may exert more favorable effects on the recovery of cardiac function in patients with large infarct size. Furthermore, it signals a potential effect of LT3 on myocardial edema and microvascular obstruction. These novel findings merit further investigation in large trials.

## 1 Introduction

Despite the progress in the therapy of acute myocardial infarction (AMI), nearly 30%–40% of patients will develop cardiac dysfunction after reperfusion with primary angioplasty (1). Early after the index event, cardiac dilatation occurs to preserve hemodynamics via Starling’s effect. Although this response is adaptive in the short term, it seems to be maladaptive in the long term. Cardiac stretch leads to pathologic growth and ultimately to cardiac dysfunction and heart failure (2). Left ventricular ejection fraction (LVEF) appears to be the strongest independent predictor of major adverse cardiovascular events (MACEs) in patients with AMI (3). Patients who experienced new-onset heart failure (HF) or death have a lower LVEF during the acute phase compared with those who did not (4). Along this line, infarct size (IS) is also one of the main determinants of prognosis in patients with AMI, with every 5% increase in infarct size contributing to a 20% increase in the relative hazard for all-cause mortality or hospitalization for HF within 1 year after primary percutaneous coronary intervention (PCI) (5).

Currently, there are no effective treatments to prevent cardiac remodeling, particularly in patients with large infarct size. Thyroid hormone is known to be a regulator of physiologic growth, which is crucial for organ maturation during development. Over the past years, numerous preclinical studies have shown that thyroid hormone can repair the postischemic myocardium by upregulating physiologic growth signaling (6–12). This novel action was recently tested in a pilot, randomized, double-blind, placebo-controlled trial (13), which showed that early triiodothyronine (LT3) administration can prevent cardiac dilatation and improve cardiac function in patients with acute myocardial infarction. Furthermore, LT3 had no effect on cardiac magnetic resonance (CMR) infarct volume at hospital discharge but facilitated infarct healing after 6 months (13).

Here, a *post-hoc* analysis of the data from the ThyRepair study was performed in order to provide more insights into the novel effects of LT3 on postinfarcted human myocardium. Specifically, we explored whether the effects of LT3 therapy on the human postinfarcted myocardium are dependent on the severity of infarct size. This analysis may be of importance for the design of future studies translating thyroid hormone into clinical practice.

## 2 Methods

### 2.1 Study design

All patients in this study were part of the original pilot, randomized, double-blind, controlled trial, ThyRepair. The study protocol, setting, and inclusion and exclusion criteria were also previously reported (13). The study (ThyRepair study, EudraCT: 2016-000631-40) was performed in accordance with the Declaration of Helsinki (revised version, 1996) and the European Guidelines for Good Clinical Practice (version 11, July 1990). The study protocol was approved by the National Independent Ethics Committee (26/16, 31-3-2016) and the Greek Drug Agency. All subjects gave written informed consent. The study was conducted in two clinical centers.

In brief, T3^®^ Solution for injection (concentration 10 μg/mL) was prepared from Uni-Pharma Pharmaceutical Laboratories S.A., Kifissia, Greece, and administered as intravenous (i.v.) bolus injection of 0.8 μg/kg of 3,3′,5-triiodo-l-thyronine sodium followed by a constant infusion of 0.113 μg·kg^−1^·h^−1^ i.v. for 48 h using a pump. Patients in the placebo group received equivalent volumes of the vehicle with identical composition apart from the active substance. Pharmacokinetic analysis of thyroid hormone levels and adverse events has been previously published (13). In the treatment group, total T3 levels were shown to increase by approximately sevenfold for 48 h compared to placebo. Serious, life-threatening events related to LT3 treatment were not observed. A modest increase in heart rate was observed in LT3-treated patients only during 72 h (13).

CMR was performed at discharge at a single center (Department of Radiology, IASO Hospital of Athens). The mean discharge time was 6.4 ± 1.5 days from admission. All MRI analyses were performed at the same core laboratory using the freely available for research purposes software Segment, version 2.0 R5201 (http://segment.heiberg.se), with the participant’s allocation status blinded. End-points of this analysis were LVEF, LV end-diastolic volume index (LVEDVi; mL/m^2^), LV end-systolic volume index (LVESVi; mL/m^2^), left ventricular mass index (LVMi), and infarct volume (IV) as assessed by CMR at discharge from hospital. The infarcted volume of LV myocardium was determined as regional delayed hyperenhancement defined as an increase in the postcontrast signal more than 2 standard deviations above that in a reference region of non-infarcted myocardium within the same slice. The following formula was used to calculate the absolute volume (in mL) of the infarcted area in each slide: hyperenhanced area (in square centimeters) × slice thickness (in centimeters). Microvascular obstruction (MVO) was defined as late hypoenhancement within a hyperenhanced region on the delayed CMR images that persisted for ≥10 min after contrast injection.

### 2.2 Statistical analysis

Summary values are expressed as mean ± SD. This is a *post-hoc* analysis that included data from 41 patients participating in the ThyRepair study (n = 20 placebo and n = 21 LT3). Patients were divided into two groups based on the median value of the infarct size expressed as a percentage of the LV: small infarct size with IV ≤ 20% of the LV (group A) and large infarct size with IV > 20% of the LV (group B).

Categorical data were compared using the chi-square and Fisher’s exact tests. The normal distribution of variables was estimated using the Shapiro–Wilk test of normality. Normally distributed data were compared using an independent t-test. Skewed data were analyzed non-parametrically using the Mann–Whitney U test.

All reported p-values are two-sided, and a value of less than 0.05 is deemed as indicative of statistical significance. Analyses were performed using the statistical software package SPSS version 23 (IBM).

## 3 Results

### 3.1 Baseline characteristics

Baseline characteristics and treatment were balanced between the placebo and LT3-treated groups (**Table 1**). No differences were observed in gender between the groups. During admission, all patients received heparin or bivalirudin, a loading dose of a P_2_Y_12_ inhibitor, and aspirin.

**Table 1:** Study population characteristics.

	Group A			Group B		
	Placebo (n = 10)	LT3 (n = 8)	p	Placebo (n = 10)	LT3 (n = 13)	p
**Age (years)**	56.6 ± 6.9	55.3 ± 7.0	0.69	57.7 ± 6.7	52.7 ± 11.7	0.25
**Hs troponin peak**	106454 ± 102788	100894 ± 60327	0.89	183129 ± 89922	200664 ± 96535	0.66
**Infarct volume, mL**	14.0 ± 11.8	16.8 ± 3.8	0.49	39 ± 10.7	35 ± 8.0	0.25
**Total ischemic time (min)**	283 ± 248	193 ± 128	0.37	315 ± 214	232 ± 178	0.32
**Proximal LAD/Mid LAD**	6 (60%)/4 (40%)	5 (63%)/3 (38%)	0.40	8 (80%)/2 (20%)	6 (46%)/7 (54%)	0.25
**3-vessel disease, no. (%)**	1 (10%)	0 (0%)	0.53	0 (0%)	1 (7.7%)	0.57
**Current smokers**	7 (70%)	4 (50%)	0.67	10 (100%)	10 (77%)	0.27
**Hypertension**	2 (20%)	1 (12.5%)	0.59	4 (40%)	7 (54%)	0.4

Values are expressed as mean ± SD.

LAD, left anterior descending artery; SD, standard deviation; p, statistical significance.

In group A, the mean age of participants was 56.6 ± 6.9 for the placebo group and 55.3 ± 7.0 years for the LT3-treated group. The total ischemic time and infarct volume were also similar in the placebo and LT3-treated groups. Peak levels of troponin were 106,454 ± 102,788 in the placebo group compared to 100,894 ± 60,327 in the T3-treated group (**Table 1**). Total T3 levels at 24 h were 0.83 ± 0.17 ng/mL for the placebo group and 5.2 ± 1.0 for the LT3-treated group, p < 0.05.

In group B, the mean age of participants was 57.7 ± 6.7 for the placebo group and 52.7 ± 11.7 years for the LT3-treated group. The total ischemic time and infarct volume were also similar in the placebo and LT3-treated groups. Peak levels of troponin were 183,129 ± 89,922 in the placebo group compared to 200,664 ± 96,535 in the LT3-treated group. Total T3 levels at 24 h were 0.78 ± 0.19 ng/mL for the placebo group and 5.7 ± 1.7 for the LT3-treated group, p < 0.05. As expected, infarct volume and peak troponin levels were significantly increased in group B as compared to group A (**Table 1**).

### 3.2 Left ventricular cardiac dilatation

**Figure 1:**
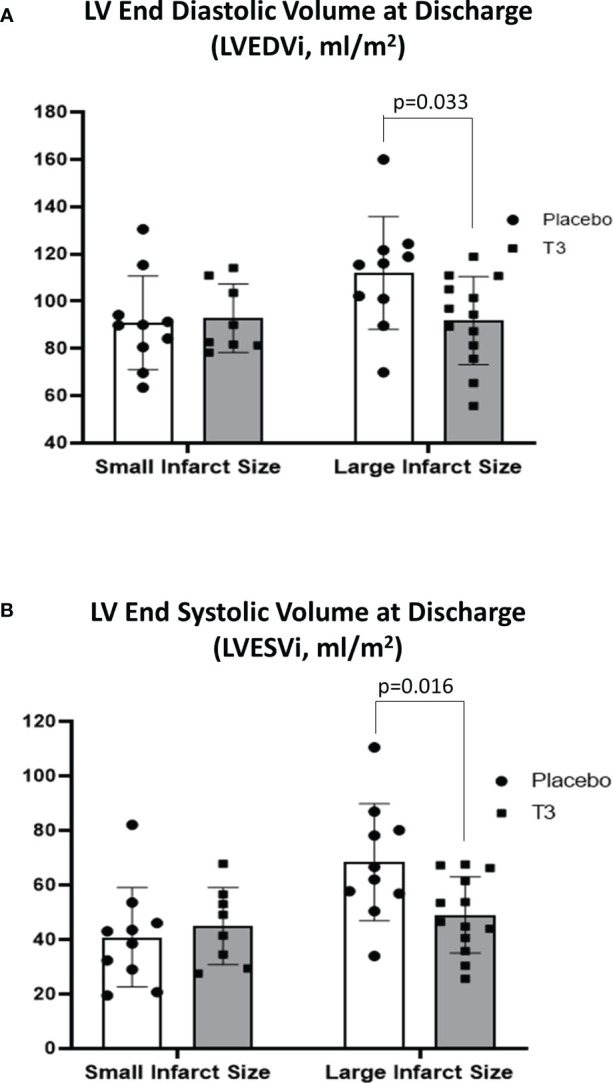
Left ventricular volumes in placebo (n = 10) and LT3-treated (n = 8) patients with small infarct size and in placebo (n = 10) and LT3-treated (n = 13) patients with large infarct size at discharge. LVEDVi **(A)** and LVESVi **(B)** are presented as scatter plots with bars showing mean values and standard deviation. LV, left ventricle; LT3, triiodothyronine; LVEDVi, LV end-diastolic volume index; LVESVi, LV end-systolic volume index.

In group A, CMR LVEDVi and LVESVi at hospital discharge were similar in both the placebo and LT3-treated groups (90.9 ± 19.8 and 40.8 ± 18.2 for the placebo group and 92.8 ± 14.5 and 44.9 ± 14.1 for the LT3-treated group) (**Figure 1**).

In group B, CMR LVEDVi was significantly lower in the LT3-treated group compared with placebo at hospital discharge (91.8 ± 18.6 vs. 112 ± 23.8, p = 0.033). CMR LVESVi was also significantly lower in the LT3-treated group compared with placebo (49.0 ± 14.0 for the T3-treated group vs. 68.3 ± 21.5 for the placebo group, p = 0.016) (**Figure 1**).

### 3.3 Cardiac function

In group A, CMR LV ejection fraction (%) at discharge was not statistically different between the placebo and T3-treated groups with small infarct size (52.2 ± 10.5 for the T3-treated group and 56.8 ± 10.2 for the placebo group).

**Figure 2:**
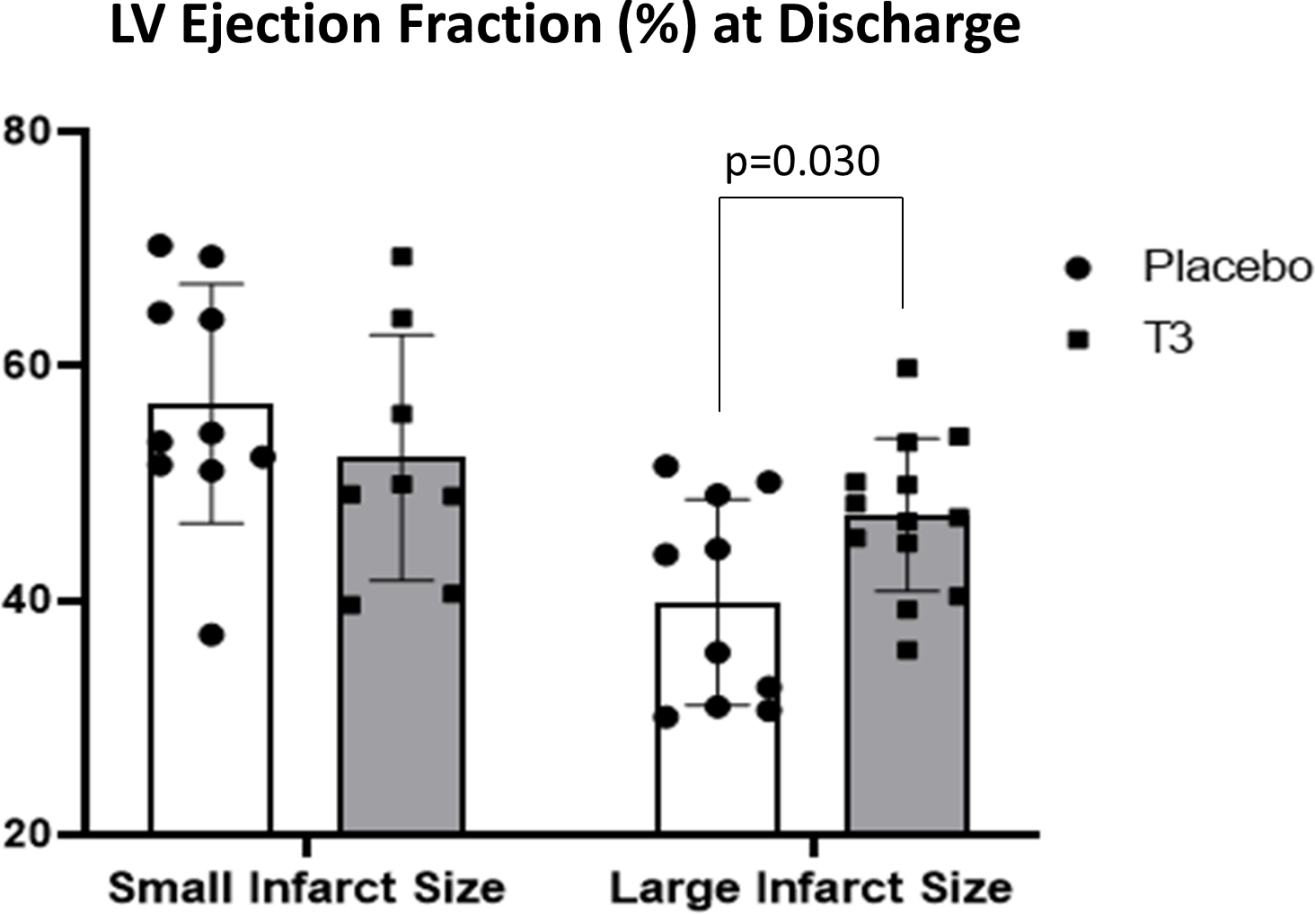
Left ventricular ejection fraction (%) in placebo (n = 10) and LT3-treated (n = 8) patients with small infarct size and in placebo (n = 10) and LT3-treated (n = 13) STEMI patients with large infarct size at discharge. LV ejection fraction (%) is presented as scatter plots with bars showing mean values and standard deviation. LV, left ventricle; LT3, triiodothyronine; STEMI, ST-elevation myocardial infarction.

In group B, CMR LV ejection fraction (%) was significantly increased in the LT3-treated group versus the placebo group (47.3 ±6.5 for the LT3-treated group vs. 39.9 ± 8.7 for the placebo, p= 0.030) (**Figure 2**).

### 3.4 Left ventricular mass and microvascular obstruction

**Figure 3:**
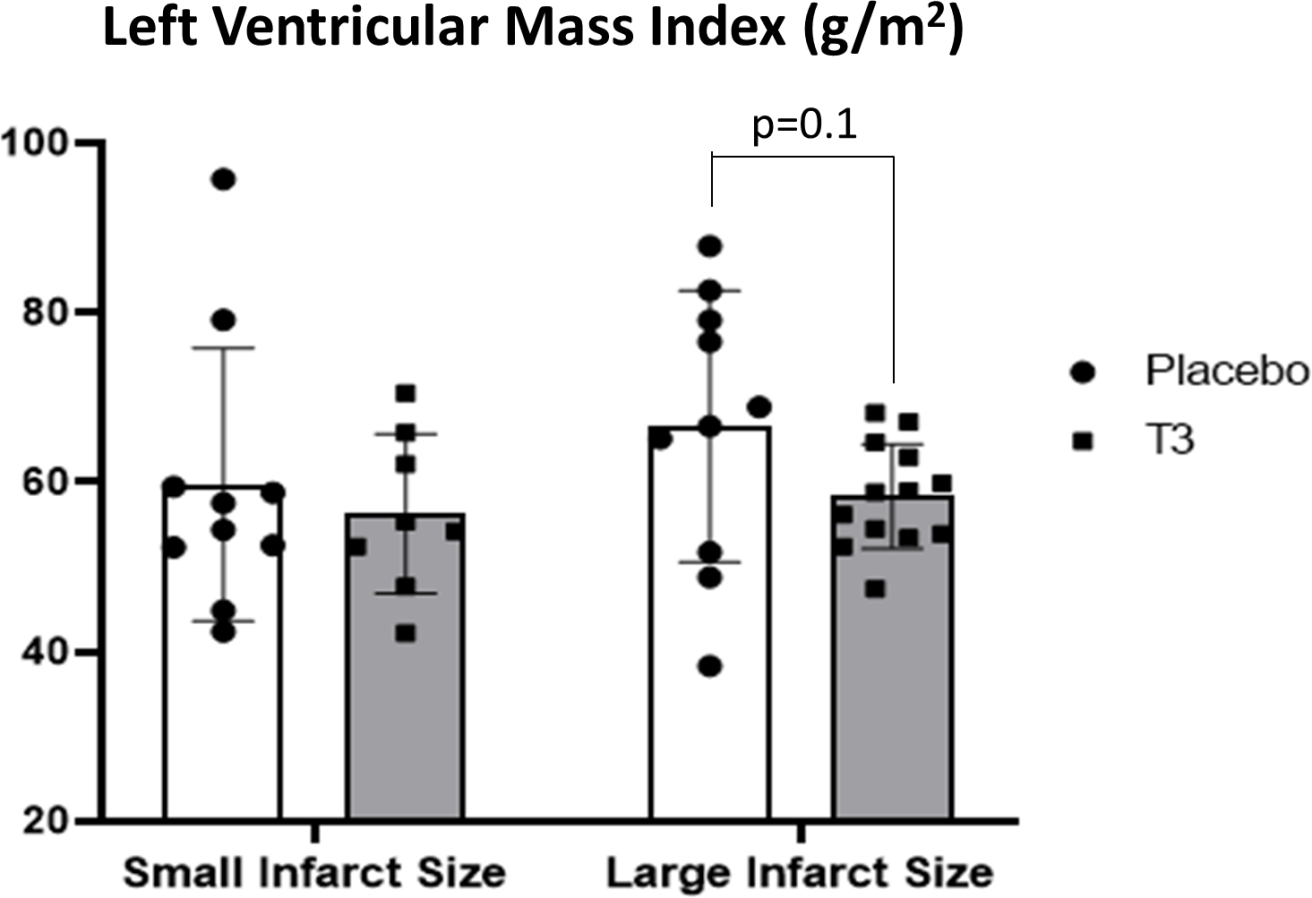
Left ventricular volumes in placebo (n = 10) and LT3-treated (n = 8) patients with small infarct size and in placebo (n = 10) and LT3-treated (n = 13) patients with large infarct size at discharge. LVEDVi **(A)** and LVESVi **(B)** are presented as scatter plots with bars showing mean values and standard deviation. LV, left ventricle; LT3, triiodothyronine; LVEDVi, LV end-diastolic volume index; LVESVi, LV end-systolic volume index.

In group A, LVMi was 59.7 ± 10.2 g/m^2^ in the placebo group vs. 56.3 ± 9.4 in the LT3-treated group, p = 0.6 (**Figure 3**). MVO was 0.58 ± 1 in the placebo group vs. 0.26 ± 0.3 in the LT3-treated group, p = 0.4.

In group B, LVMi was 66.6 ± 8.7 g/m^2^ in the placebo group vs. 58.3 ± 6.2 in the LT3-treated group, p = 0.1 (**Figure 3**). MVO was 1.95 ± 2.2 in the placebo group vs. 0.84 ± 0.9 in the LT3-treated group, p = 0.15.

## 4 Discussion

ThyRepair is the first of its kind pilot, randomized, double-blind, placebo-controlled trial that investigated the effects of acute, high-dose LT3 treatment in patients with anterior myocardial infarction. LT3 was administered immediately after reperfusion and for 48 h thereafter. LT3 was used in high doses according to dose-dependent studies in animal models of myocardial ischemia–reperfusion (13). This therapeutic regimen resulted in favorable effects on the recovery of cardiac function without major safety issues (13).

The effects of LT3 treatment were assessed by CMR. CMR is considered the gold standard imaging modality for quantitative and qualitative assessment of myocardial infarct size, left ventricular volumes, and ejection fraction (14). CMR LVEF% was the primary end-point of the ThyRepair study; CMR cardiac volumes, infarct volume, LVMi, and MVO indices were the secondary end-points. CMR LVEF% was found to be higher in LT3-treated patients, and although the magnitude of the difference compared to placebo was of clinical importance, it did not reach statistical significance (13). This was attributed to an inadequate number of patients included in the study and the early functional compensatory mechanisms that occur at an early stage after myocardial infarction (13).

In the present study, we performed a *post-hoc* analysis in order to explore whether the effects of LT3 therapy on the human postinfarcted myocardium are dependent on the severity of infarct size. This may offer a possible explanation for the variation in the recovery of CMR LVEF% after LT3 treatment, as seen in the ThyRepair Study. To address this issue, we divided the patients into two groups: one group included patients with large infarct size with CMR infarct volume above 20% of left ventricular volume, and the other group had small infarct size with CMR infarct volume less than or equal to 20% of left ventricular volume. Baseline characteristics, such as age, total ischemic time, and co-morbidities, were not significantly different between LT3-treated and placebo patients in either the small infarct or large infarct group. Total ischemic time had a trend to be lower in LT3-treated patients, but this difference was not statistically significant. There is much controversy over the use of this index in clinical practice because reporting bias is an inherent limitation of studying symptom-to-balloon time. Patients may find it difficult to estimate the time of symptom onset, particularly in the acute setting of illness. Furthermore, the optimal time still has not been defined (15). Thus, troponin release and CMR infarct volume are more accurate to estimate the ischemic damage. Both indices were similar between LT3-treated and placebo patients in either the small infarct or large infarct group. To our surprise, LT3 resulted in significant improvement of all CMR functional indices (LVEF% and cardiac volumes) only in patients with large infarct size. This finding probably indicates that LT3 treatment can potentially benefit the group of patients with large infarct size and worse prognosis (5). However, taking into consideration the small number of patients included in the analysis, this issue merits further investigation.

The use of thyroid hormone in the setting of myocardial ischemia has been a controversial issue over the past years. Thyroid hormone increases heart rate and oxygen consumption and could potentially be detrimental in the setting of ischemia. However, in the era of reperfusion, the role of thyroid hormone in ischemia–reperfusion injury has been redefined. During reperfusion, despite restoration of oxygen supply, glucose oxidation is suppressed and acidosis occurs, which leads to calcium overload and cardiac dysfunction. Triiodothyronine treatment has been shown to improve the coupling of glycolysis to glucose oxidation via its action on pyruvate dehydrogenase (PDH), reduce acidosis, and improve functional recovery (16, 17). Interestingly, the triiodothyronine effect on postischemic cardiac function has not been associated with increased myocardial oxygen consumption (16) and increased myocardial injury in animals (18) or in patients with bypass surgery, acute myocardial infarction, or chronic heart failure (13, 19, 20).

This *post-hoc* analysis of the ThyRepair data has also provided some novel mechanistic insights into the potential effect of LT3 on early myocardial edema and microvascular obstruction. This evidence may be of therapeutic relevance since effective treatments for MVO and edema are not currently available. The present analysis showed that in patients with large infarct size, LT3 treatment resulted in lower CMR LVMi and MVO. Although these differences did not reach statistical significance, they seem to be of physiological importance. LV mass increases acutely after myocardial infarction due to accumulation of myocardial edema from ischemia–reperfusion injury resulting in increased wall thickness and appears as increased LVMi in CMR imaging. Myocardial edema is not only a consequence of severe ischemia–reperfusion insult but, in turn, can contribute to impairment of microcirculation during reperfusion by mechanically compressing the microvasculature seen as increased MVO in CMR imaging (21). Obstruction of the microcirculation prevents the healing process and exacerbates remodeling after myocardial infarction. Thus, the presence of MVO following myocardial infarction is associated with reduced LV function, greater cardiac remodeling, and increased cardiac events such as heart failure and death (22). Therefore, potential treatments for preventing cardiac remodeling need to be initiated early after primary angioplasty. This is a plausible explanation for the failure of thyroid hormone treatment to show favorable effects on cardiac remodeling when administered at later stages of myocardial infarction (23, 24). Taken together, it seems that early LT3 treatment may have beneficial effects on myocardial edema and MVO, which merit further investigation in larger trials. The vast majority of past and current MVO trials have targeted the intravascular component of MVO using intracoronary vasodilators (adenosine, sodium nitroprusside, and calcium channel blockers) or antithrombotics with mixed results. Targeted intracardiac hypothermia is the only therapy that is currently investigated in a clinical trial as a potential treatment for myocardial edema (25).

## 5 Conclusion

The present study, based on *post-hoc* analysis of data obtained from the ThyRepair study, indicates that acute LT3 treatment may exert more favorable effects on the recovery of cardiac function in patients with large infarcts. Furthermore, it signals a potential effect of LT3 on myocardial edema and microvascular obstruction. However, the small number of patients included in this study has to be taken into consideration, and these data need to be validated in large clinical trials.

## Author’s note

These authors take responsibility for all aspects of the reliability and freedom from bias of the data presented and their discussed interpretation.

## Data availability statement

The raw data supporting the conclusions of this article will be made available by the authors, without undue reservation.

## Ethics statement

The studies involving humans were approved by the National Independent Ethics Committee (26/16, 31-3-2016) and the Greek Drug Agency. The studies were conducted in accordance with the local legislation and institutional requirements. The participants provided their written informed consent to participate in this study.

## Author contributions

CP: Writing – review & editing, Writing – original draft, Project administration, Formal analysis, Conceptualization. KG: Data curation, Formal analysis, Investigation, Methodology, Supervision, Writing – original draft, Writing – review & editing. AT: Writing – review & editing, Supervision, Methodology, Investigation, Data curation. NA: Data curation, Methodology, Writing – review & editing. IM: Conceptualization, Formal analysis, Supervision, Validation, Writing – original draft, Writing – review & editing.
